# A new Colombian species of *Liodessus* diving beetles from the Páramo de Sumapaz (Coleoptera, Dytiscidae, Bidessini)

**DOI:** 10.3897/zookeys.1059.70134

**Published:** 2021-09-08

**Authors:** Michael Balke, Yoandri Suarez-Megna, Rodulfo Ospina-Torres, Juan Simon Venegas, Carlos Prieto, Lars Hendrich

**Affiliations:** 1 SNSB-Zoologische Staatssammlung München, Münchhausenstraße 21, D-81247 Munich, Germany SNSB-Zoologische Staatssammlung München Munich Germany; 2 GeoBioCenter, Ludwig Maximilians University, Munich, Germany Ludwig Maximilians University Munich Germany; 3 Departamento de Biología, Universidad de Oriente. Patricio Lumumba s/n, Santiago, Santiago de Cuba, Cuba Universidad de Oriente Santiago de Cuba Cuba; 4 Departamento de Biologıa, Universidad Nacional de Colombia, Bogota, Colombia Universidad Nacional de Colombia Bogota Colombia; 5 Departamento de Biología, Universidad del Atlántico, Barranquilla, Colombia Universidad del Atlántico Barranquilla Colombia; 6 Corporación Universitaria Autónoma del Cauca, Popayán, Colombia Corporación Universitaria Autónoma del Cauca Popayán Colombia

**Keywords:** Colombia, Dytiscidae, *
Liodessus
*, new species, Páramo, Sumapaz

## Abstract

*Liodessuspicinus***sp. nov.** is described from the Páramo de Sumapaz near Bogota D.C. at 3,500 m above sea level. The species can be distinguished from the other Colombian *Liodessus* species by its dark coloration, discontinuous habitus, shiny surface of the pronotum and elytron, presence of a distinct occipital line, distinct basal pronotal striae, short or even faint basal elytral striae, as well as by its distinct geographic distribution and cox1 signature.

## Introduction

Diving beetles of the genus *Liodessus* Guignot, 1939 belong to the tribe Bidessini and occur in the New World as well as the Afrotropical Region (Biström 1988; Nilsson and Hájek 2021). They are typically smaller than 3 mm and inhabit a variety of mainly lotic habitats. Andean species reach altitudes of nearly 5,000 m, where they are the most abundant aquatic beetles (Balke et al. 2020a, 2020b).

However, diving beetles from the high altitudes of the Puna and Páramo regions remain poorly studied. Since 2019, as the result of a research and training cooperation between our institutions, 10 new species were described from these regions of Peru (Balke et al. 2019, 2020b) and Colombia (Megna et al. 2019; Balke et al. 2021). It became apparent that many more new species of *Liodessus* remain to be discovered in the vast Andean highland ecosystems, most of them likely endemic to one or a few Páramo or Puna areas, respectively. To address this in a combined evidence pipeline, we suggested a DNA sequence-based platform for the study of these insects (Balke et al. 2020a, 2020b) using the Barcode of Life Data System (BOLD) of the Canadian Centre for DNA Barcoding and the 5′ mitochondrial *cox1* gene fragment (http://www.boldsystems.org) (Ratnasingham and Hebert 2007).

It is well understood that one genetic marker alone cannot be the omnipotent tool for taxonomy, in particular markers such as *cox1*, which are not involved in speciation *per se* (Kwong et al. 2012). Rather, such DNA sequence data have the purpose of guiding the sorting of specimens to operational units and then flank taxonomic decision-making process. This approach has been utilized successfully to study very diverse beetle taxa (Tänzler et al. 2012; Riedel et al. 2013a, 2013b), including our own previous work on *Liodessus*. The approach can technically be scaled up massively using next generation sequencing technology, which is already also reducing analytical costs (Wang et al. 2018). This does, however, and that is important to note, not replace taxonomic expertise and the evaluation of morphological structures (Riedel et al. 2013a). Here, we report the discovery of another new species of *Liodessus* from Páramo de Sumapaz (Fig. 3A), the world’s largest Páramo system, near Bogota, Colombia. Interestingly, while we sampled more than 100 specimens of *Liodessusbogotensis* Guignot, 1953, we only found three females of the new species.

## Materials and methods

### Acronyms

**LIAUN** Laboratorio de Insectos Acuáticos, Departamento de Biología, UniversidadNacional de Colombia, Bogota, Colombia;

**ZSM**SNSB-Zoologische Staatssammlung, München, Germany; temporarily stored for further morphological work.

### Morphological descriptions and photography

The description of morphological characters follows our previous work on *Liodessus* beetles (e.g. Balke et al. 2020b).

Images were taken with a Canon EOS R camera. We used a Mitutoyo 10× ELWD Plan Apo objective, attached to a Carl Zeiss Jena Sonnar 3.5/135 MC as focus lens. Illumination was with three LED segments SN-1 from Stonemaster (https://www.stonemaster-onlineshop.de). Image stacks were generated using the Stackmaster macro rail (Stonemaster), and images were then assembled with the computer software Helicon Focus v. 4.77TM on an iMac with a Radeon Pro 5500 XT GPU.

### DNA analysis

The DNA sequencing and data analysis laboratory protocol follows standard Canadian Centre for DNA Barcoding (CCDB) barcoding procedures (https://ccdb.ca/). We delivered tissue samples to CCDB, which were processed, and the barcode data uploaded to BOLD systems. We used a simple approach to calculate a neighbour-joining tree (*p*-distances) in Geneious software v. 11.0.4 in order to learn if newly added entries could be assigned to existing species groups or not. This approach has been proven helpful and strongly guiding the morphological descriptive process, not the least by enabling us to unambiguously identify the new species presented here in the absence of male specimens.

## Results

### Liodessus picinus sp. nov.

Taxon classificationAnimaliaColeopteraDytiscidae

41D32606-0C70-536D-806C-D1C8C0810C40

http://zoobank.org/AD2344A0-BD40-47BB-B99D-6B20430DC8A1

Figures 1 , 2G , 3

#### Type locality.

Sumapaz National Park, Bogota, Colombia.

#### Holotype.

“Colombia, ♀; Bogota D.C., PN Sumapaz; 3,500 m; 13.xi.2018; 4.290°N; 74.207°W; Ospina, Venegas, Balke and Megna (COL_MB_2018_04) Voucher MB8416” (LIAUN).

#### Paratypes.

2 ♀♀, same data but voucher numbers MB8414, 8415 (ZSM).

#### Description of holotype.

**Figure 1.**
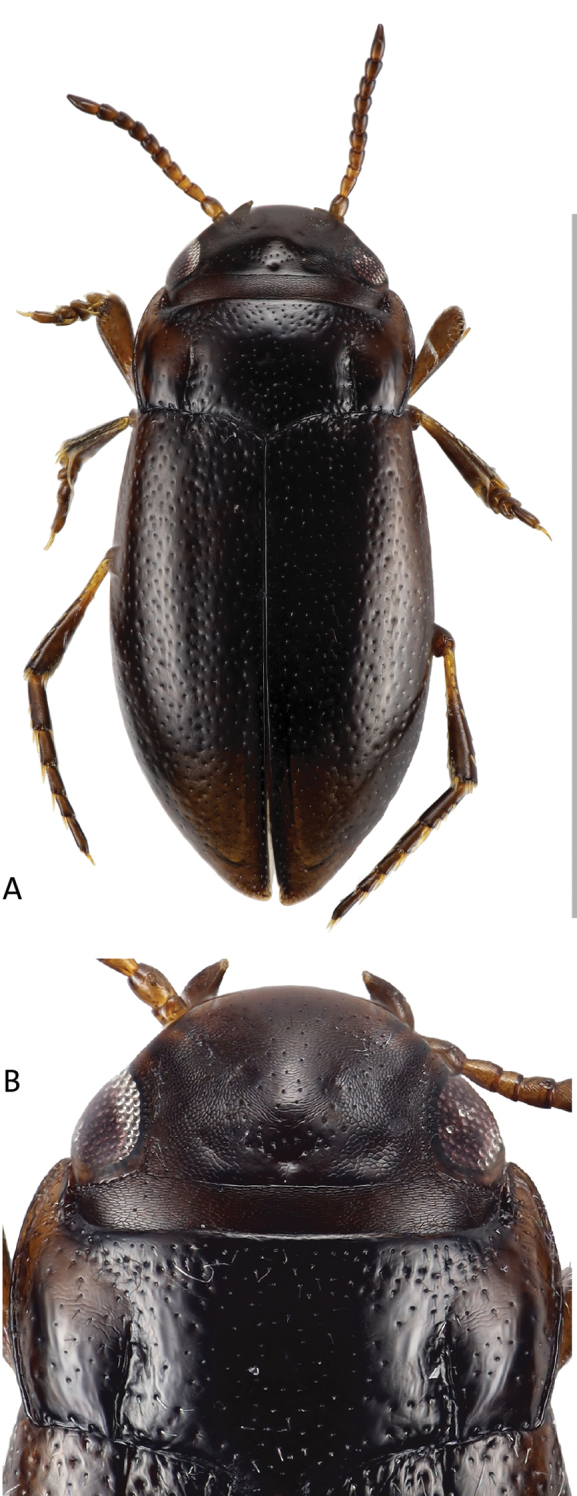
*Liodessuspicinus* sp. nov. **A** dorsal habitus **B** dorsal head, pronotum, and base of elytra. Scale bar: 2 mm.

Habitus with distinct discontinuity between pronotum and elytra (Fig. 1A), pronotum widest before base (Fig. 1A, B). Total length of beetle 2.0 mm; length without head 1.8 mm; maximum width 0.9 mm.

**Color.** Very dark brown to blackish dorsally and ventrally, base of meso- and metatibia contrastingly of lighter color (Fig. 1A, B).

**Surface sculpture.** Head with few setiferous punctures in front of a distinct occipital line, distinct microreticulation present except on middle of head between the eyes (Fig. 1B); posteriorly of occipital line with distinct microreticulation and few punctures. Pronotum and elytron shiny, with moderately dense and coarse setiferous punctation; pronotum with few wrinkles laterally (Fig. 1B).

**Structures.** Head with distinct occipital line, with rounded clypeus. Antenna stout. Pronotum with distinct lateral bead and distinct, long and deep basal striae (Fig. 1A, B). Elytron with short basal striae, without sutural line and without basal epipleural transverse carina. Metathoracic wings not examined in holotype (in paratype: short, about half the length of elytron).

#### Variation.

One paratype is slightly larger, TL 2.1 mm. In one paratype, the elytral basal striae are not very obvious.

#### Male.

Unknown.

#### Etymology.

Picinus, black, highlighting the dark coloration of the species.

#### Comparative notes.

The species is well characterized by its small size (2.0–2.1 mm total length, shorter than most other Colombian Páramo species (Megna et al. 2019; Balke et al. 2021) except *L.lacunaviridis* (Balke et al. 2021)); dark coloration, discontinuous habitus, shiny surface of pronotum and elytrae, presence of distinct occipital line, distinct basal pronotal striae with short or even faint basal elytral striae.

We assembled sequences of the Colombian species, trimming the sequence ends to avoid missing characters. We obtained a 611 base pair reference alignment to observe sequence variation. *Liodessuspicinus* sp. nov. differs by approx. 9–11% (5′ *cox1*) from each of the other Colombian species and therefore forms a well-delineated clade with distinct mitochondrial *cox*1 signature.

**Figure 2.**
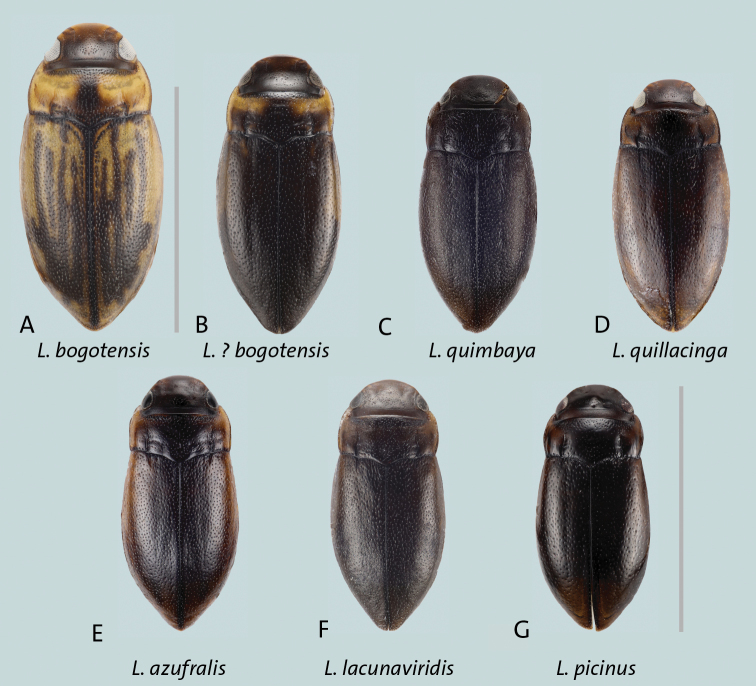
*Liodessus* spp. Dorsal habitus without appendages **A***L.bogotensis***B***L.bogotensis* darker form from Sumapaz **C***L.quimbaya***D***L.quillacinga***E***L.azufralis***F***L.lacunaviridis***G***L.picinus* sp. nov. Scale bars: 2 mm.

Distinguishing characters of the other Colombian Páramo species from the new species are:

*Liodessusazufralis*Megna et al., 2019: lighter coloration, brown to dark brown (Fig. 2E);

*L.bogotensis*: brighter coloration (Fig. 2A, B); *L.lacunaviridis*Balke et al., 2021 and *L.quimbaya*Megna et al., 2019: female with dull surface of pronotum and elytron due to presence of fine microreticulation (e.g. Fig. 2C, F); *L.quillacinga*Megna et al., 2019: long, distinct basal elytral stria (Fig. 2D).

#### Distribution.

Only known from the type locality.

#### Habitat.

**Figure 3.**
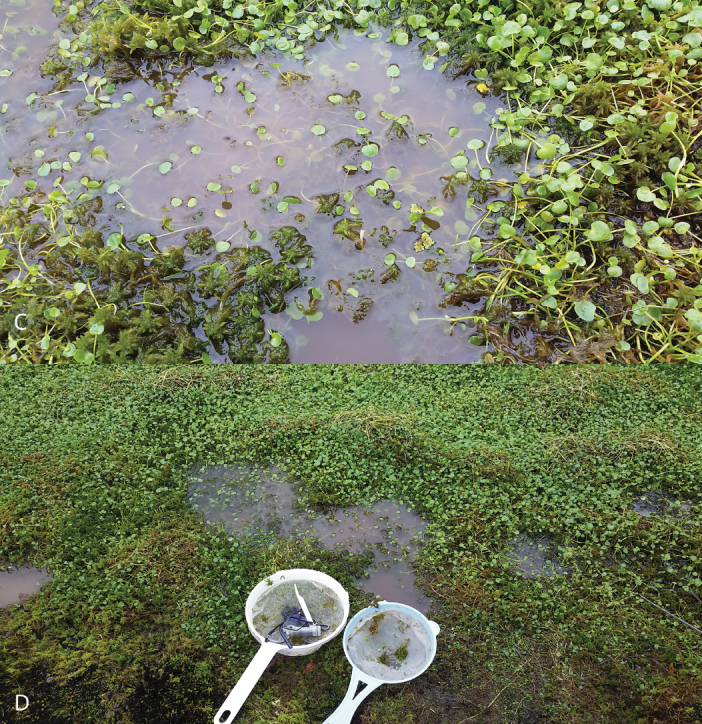
Habitat of *Liodessuspicinus* sp. nov. **A** Sumapaz National Park, general setting **B–D** collecting site with collecting tools.

Shallow and exposed peatland puddles, collected with strainer out of mats of vegetation including mosses (most likely *Sphagnum* sp.), *Ranunculus* sp. and *Azolla* sp. (Fig. 3B–D). The species is syntopic with *L.bogotensis*.

##### Checklist of the High Andean species of *Liodessus* Guignot, 1953

1. *Liodessusacollensis* Guignot, 1955: Peru

2. *Liodessusalpinus* Balke, Megna, Zenteno, Figueroa & Hendrich, 2020b: Peru

3. *Liodessusaltoperuensis* Balke, Megna, Zenteno, Figueroa & Hendrich, 2020a: Peru

4. *Liodessusandinus* Guignot, 1957: Bolivia

5. *Liodessusazufralis* Megna, Hendrich & Balke, 2019: Colombia

6. *Liodessusbogotensis* Guignot, 1953: Colombia

7. *Liodessuscaxamarca* Balke, Megna, Zenteno, Figueroa & Hendrich, 2020a: Peru

8. *Liodessushauthi* Balke, Megna, Zenteno, Figueroa & Hendrich, 2020b: Peru

9. *Liodessuslacunaviridis* Balke, Ospina-Torres, Megna & Hendrich, 2020a: Colombia

10. *Liodessuspicinus* sp. nov.: Colombia

11a. *Liodessusquillacingaquillacinga* Megna, Hendrich & Balke, 2019: Colombia

11b *Liodessusquillacingacochaensis* Megna, Hendrich & Balke, 2019: Colombia

11c. *Liodessusquillacingacumbalis* Megna, Hendrich & Balke, 2019: Colombia

12. *Liodessusquimbaya* Megna, Hendrich & Balke, 2019: Colombia

13. *Liodessusrhigos* Balke, Megna, Zenteno, Figueroa & Hendrich, 2020b: Peru

14. *Liodessusthespesios* Balke, Megna, Zenteno, Figueroa & Hendrich, 2020b: Peru

## Supplementary Material

XML Treatment for Liodessus picinus
